# Pulmonary embolism and 529 human blood metabolites: genetic correlation and two-sample Mendelian randomization study

**DOI:** 10.1186/s12863-022-01082-6

**Published:** 2022-08-29

**Authors:** Ruoyang Feng, Mengnan Lu, Jiawen Xu, Feng Zhang, Mingyi Yang, Pan Luo, Ke Xu, Peng Xu

**Affiliations:** 1 Department of Joint Surgery, HongHui Hospital, Xi’an Jiaotong University, Xi’an, 710054 Shanxi China; 2 Department of Pediatrics, The Second Affiliated Hospital of Xi’an Jiaotong University, Xi’an, Shaanxi China; 3 Orthopedic Research Institute, Department of Orthopedics, West China Hospital, Sichuan University, 37# Guoxue Road, Chengdu, 610041 People’s Republic of China; 4 Key Laboratory of Trace Elements and Endemic Diseases, National Health and Family Planning Commission, School of Public Health, Health Science Center, Xi’an Jiao tong University, No.76 Yan Ta West Road, Xi’an, 710061 People’s Republic of China

**Keywords:** Pulmonary embolism, Human blood metabolites, Genetic correlation, Genome-wide association study

## Abstract

**Background:**

The incidence of pulmonary embolism complications in the literature ranges from 10 to 50%, with a 0.5–10% risk of fatal pulmonary embolism. However, the biological cause of pulmonary embolism is unknown.

**Methods:**

This study used data from the Genome-Wide Association Study (GWAS) of Pulmonary Embolism and Human Blood Metabolites from the UK Biobank, and the data from subjects of European ancestry were analyzed. We explored the relationship between pulmonary embolism and blood metabolites in three ways. We first analyzed the genetic correlation between pulmonary embolism and human blood metabolites using the linkage disequilibrium score regression (LDSC) and then analyzed the causal relationship between pulmonary embolism and meaningful blood metabolites obtained from the LDSC, a procedure for which we used Mendelian randomization analysis. Finally, we obtained transcriptome sequencing data for patients with a pulmonary embolism from the GEO database, analyzed differentially expressed genes (DEGs) in patients with pulmonary embolism versus healthy populations, and compared the DEGs with the resulting blood metabolite genes to further validate the relationship between pulmonary embolism and blood metabolites.

**Result:**

We found six human blood metabolites genetically associated with pulmonary embolism, stearic acid glycerol phosphate ethanolamine (correlation coefficient = 0.2582, *P* = 0.0493), hydroxytryptophan (correlation coefficient = 0.2894, *P* = 0.0435), and N1-methyladenosine (correlation coefficient = 0.0439, *P* = 0.3728), and a significant causal relationship was discovered between hydroxytryptophan and pulmonary embolism. After screening microarray data from the GEO database, we performed differential gene analysis on the GSE19151 dataset and screened a total of 22,216 genes with *P* values less than 0.05, including 17,361 upregulated genes and 4854 downregulated genes. By comparing the resulting differentially expressed genes with six genes encoding blood metabolites, LIPC and NAT2 were found to be differentially expressed in association with pulmonary embolism.

## Introduction

Venous thromboembolism (VTE), consisting of deep vein thrombosis (DVT) and its complication, pulmonary embolism (PE), is a major health problem [1]. PE is a clinical and pathophysiological syndrome caused by an endogenous or exogenous embolus blocking the pulmonary artery trunk or branches, resulting in obstruction of the pulmonary circulation [2]. PE has the third highest incidence after coronary heart disease and hypertension and the third highest mortality rate after cancer and my ocardial infarction. Acute PE is a major public health problem, causing 100,000–180,000 deaths annually in the United States [2, 3].There are studies that show that patients with hip fractures are at high risk of VTE, including DVT and PE, which is a major cause of morbidity and mortality [4].There are many complications after bone fractures, of which pulmonary embolism is one of the highest mortality complications. The incidence of complications of pulmonary embolism after bone fracture surgery, with a high mortality rate, has become a common concern among medical staff. When bone fractures are complicated, the main cause of pulmonary embolism is blood stasis, the hypercoagulable state of blood and damage to the vascular wall, and PE is also related to factors such as prolonged bed rest and major surgery. The pathogenesis is unknown but likely involves environmental and genetic factors [5].

Human blood metabolites are the products of human blood metabolism and vary from person to person. Already detailed information of numerous metabolites found in human biospecimens, such as blood, urine or cerebrospinal fluid is deposited in the Human Metabolome Database [6]. Blood metabolites, which often reflect the genetic makeup of individuals, serve as functional intermediates after environmental exposure and can predict or influence the development of disease [7]. Therefore, the analysis of metabolites in blood, also called blood metabolome, is a promising tool for early stage diagnosis and continuous health-status monitoring [8]. Because they are easy to sample, the biological significance and function of noncellular metabolites (plasma or serum) are often analyzed [9]. Genome-wide affiliation considers of metabolite concentrations (mGWAS) can give novel bits of knowledge into human physiology, innate mistakes of digestion system, and complex characteristics and illnesses, and most past mGWAS have centered on blood metabolite concentrations in population-based studies [10]. Past ponders have investigated the relationship between human blood metabolites and infections such as diabetes, osteoporosis, and cancer [9–11]. Bujak et al. analyzed metabolic plasma profiles gotten from a porcine pneumonic embolism show and found changes within the levels of numerous metabolites included in glycolysis, tricarboxylic corrosive (TCA) cycle intermediates, lipid digestion system, and ketone bodies [12]. A ponder by Zeleznik et al. in patients with distinctive seriousness of PE showed critical contrasts within the tricarboxylic corrosive cycle, greasy corrosive and purine metabolite pathways in patients with moo- and intermediate−/high-risk PE [13]. The field of metabolomics has as of late been broadly created and utilized to distinguish potential biomarkers for early determination of infections. Metabolite thinks about can uncover the physiological state earlier to illness onset and the complex intuitive between qualities, natural chance variables and venous thromboembolism [14]. This technique has been used in the study of cardiovascular disease risk, however, there are only a few publications on the metabolic analysis of PE, thus the results of this study are a great contribution to the field of pulmonary embolism metabolism.

Genetic correlations are correlations between phenotypes of hybrid populations due to genotype. Interpretation of GWAS data is frequently confounded by LDSC [15]. Recently, a novel technique to estimate genetic correlation between two traits, LDSC was developed [16]. LDSC quantifies the contribution of each factor by testing the relationship between the statistic and LDSC [17]. However, the use of LDSC alone often provides only a genetic association between human blood metabolites and the occurrence of pulmonary embolism, while the causal relationship between the two is unclear. Mendelian randomization (MR), a popular method to study genetic epidemiology, allows the exploration of causal relationships between exposure and outcome by using genetic variation as an instrumental variable [18]. MR is an analysis that uses genetic variants, which are expected to be independent of confounding factors, as instrumental variables to test for causality [19]. MR is very convincing in verifying the causal relationship between exposure variables and outcome variables [20], especially when the two have been shown to be genetically correlated. LDSC regression and MR analyses could further dissect the associations between inflammation, metabolic factors, and depressive symptoms [21]. We also employ statistical genetics methods such as linkage disequilibrium LDSC and MR. MR has evolved as a valuable tool for investigation of causal relationships between risk factors and complex traits [22].

We employed LDSC and MR analyses to reveal the genetic correlation and causal association between pulmonary embolism and 453 blood metabolites. Moreover, we also conducted reverse MR to look at the bidirectional causal effect of blood metabolites on pulmonary embolism.

This study reveals for the first time that genome-wide association analysis data between pulmonary embolism and human blood metabolites have been used for analysis. The genetic correlation and causal association between pulmonary embolism and 453 blood metabolites were revealed by chain imbalance regression scoring and Mendelian randomization analysis, and were compared with differential genes obtained from mRNA expression profiling to further illustrate the genetic association. The genetic association of pulmonary embolism with human blood metabolites was analyzed in this study at the DNA level using LDSC as well as Mendelian randomization, and we further validated the co-expression of differential genes for pulmonary embolism with their blood metabolite counterparts at the mRNA level using transcriptome sequencing data from the GEO database. These data have not been previously analyzed in this way or discussed. Therefore, this study may provide strong evidence about the mechanism of pulmonary embolism formation, provide new ideas and insights to prevent pulmonary embolism after bone fracture surgery, and provide new ideas for the clinical treatment of pulmonary embolism.

## Methods

### The GWAS summary data of pulmonary embolism

Data for the genome-wide association study of pulmonary embolism were obtained from published studies from the UK Biobank [23]. These data were studied in a population from Europe comprising a total of 452,264 study subjects, including 448,312 control and 3952 experimental populations. Disease-related information was collected from each participant and blood samples were taken during the subject’s visit to the UK Biobank Assessment Center, and DNA extraction and genotyping were performed at the Affymetrix Research Services laboratory. There were 62,394 genotyped variants by applying quality control of the Biosystems UK Biobank axiomatic array containing 9,113,133 screened interpolated variants. The IMPUTE4 program was used to perform the interpolation (http://jmarchini.org/software/). A detailed description of the study sample characteristics, study design and statistical analysis can be found in Biobank UK [23].

### GWAS data of human blood metabolites

Human blood metabolites were downloaded from a published study [24]. Shin et al. sequenced the whole genome of 7824 adults from Twins UK and Kora databases in Europe to assess the relationship between genetic variation and blood metabolism. The data from this study contained a total of 2.1 million SNP loci and 486 blood metabolites (309 known metabolites and 177 unnamed metabolites) for genome-wide association analysis. These metabolites can be broadly classified into 8 major groups: carbohydrates, amino acids, nucleotides, cofactors and vitamins, lipids, peptides, energy products, and heterologous biological metabolites. Analysis was performed by liquid chromatography, gas chromatography and coupled t and mass spectrometry. Following the QC step, all 486 metabolite concentrations in the TwinsUK and Kora datasets were initially correlated with each SNP (based on the HAPMAP2 input genotype dataset) using linear regression models from merlin and QuickTest of Tware, respectively. After quality control, genetic correlation analysis was performed for the 486 blood metabolites of 486. A detailed description of the test characteristics involved in the experiment, quality control and statistical analysis can be found in the published study [24].

### Genetic correlation

We performed LDSC analysis of the genetic correlation between pulmonary embolism and human blood metabolites using software (https://github.com/bulik/ldsc) [25], according to the standard algorithm recommended by the investigators. LDSC quantifies the contribution of each factor by testing the relationship between the statistic and LDSC. LDSC analysis concluded that for a polygenic trait, high heritability markers increased the χ2 statistic for SNPs compared to low heritability markers, and single SNP heritability was significantly increased for low heritability markers [26]. Therefore, LDSC can be used to distinguish polygenic effects from confounding biases such as ambiguous correlations and population stratification in GWAS [17]. After different tests and corrections, the threshold of significance in this study should be *P* < 1.38 × 10–5 after correction for multiple tests (0.05/3622 = 1.38 × 10–5).

### Assessing the causal relationship between pulmonary embolism and blood metabolites

We performed analyses using a two-sample Mendelian randomization model with human blood metabolites as exposure variables and pulmonary embolism as outcome variables. For Selection of IVs we used standard parameters (aggregation window of 10,000 kb, r2 cutoff 0.001) to discard variants in linkage disequilibrium (LD) with thresholds set at *p* < 5 × 10–8 for all snps. To avoid potential weak instrumental bias, the F statistic (F = beta2/se2) was used to assess the strength of IV. If F > 10, the correlation between IV and exposure is considered to be strong enough that the results of the MR analysis can be protected from weak instrumental bias [27]. The total variance explained by SNPs in these IVs was 13.9%, and the mean and total F-statistics were 115.92 and 1864.55, respectively, indicating a strong IV. In this study, inverse variance weighting (IVW) model was used as the primary causal effect estimate [20]. We performed a two-sample MR analysis using the fixed-effects IVW method, which is the most widely used method in MR studies and provides reliable causal estimation validity in the absence of directionality. Although this method has excluded known confounding SNPs as much as possible, there are still many unknown factors that can lead to genetic pleiotropy and confound the estimation of effect values. Therefore, we also used an alternative method to check the reliability and stability of the results. We also discussed and corrected for diversity of the results using both the MR-Egger regression method [28] and the weighted median [29]. MR-Egger regression analysis model was used to assess the directional multiplicity of the instruments, while the weighted median is at an advantage because it maintains higher precision over the range of estimates compared to MR-Egger analysis. The three key assumptions of MR are as follows: 1) genetic instruments are strongly associated with exposure of interest; 2) confounders of exposure-outcome associations are independent of genetic instruments; and 3) except for the association with exposure, genetic instruments are not associated with outcome [20]. In this study, we estimated the effect size through the instrumental variable method, so the study satisfies the fourth hypothesis: the effect of exposure on the outcome is homogeneous [30]. The principal analyses for pulmonary embolism were conducted using inverse-variance weighted meta-analysis and excluded potential outlier SNPs (*P* < 0.10) identified using the MR Pleiotropy RESidual Sum and Outlier (MR-PRESSO) model. Both discovery and replication results were meta-analysed using the inverse variance model, and the combined result was filtered again on Bonferroni adjusted *p*-value of *p* < 5.08 × 10–13 and heterogeneity (*p* ≥ 0.001). All MR analyses were performed using the MR-Base platform (http://app.MRBase.html.org/), and we considered the results statistically significant when *P* < 0.05.

### Screening for differentially expressed genes associated with pulmonary embolism

The GEO database name (https://www.ncbi.nlm.nih.gov/GEO/) Gene Expression Omnibus, created and maintained by the National Center for Biotechnology Information (NCBI) Gene Expression Database, is a public repository for a variety of high-throughput experimental data, including high-throughput gene expression data submitted by research institutions around the world. We downloaded GSE19151 microarray data containing 62 pulmonary embolism samples and 71 normal samples from the GEO database for differential gene analysis using GEO2R (https://www.ncbi.nlm.nih.gov/geo/geo2r/). We compared differentially expressed genes with blood metabolite genes to further validate the co-expression of differential genes in pulmonary embolism with blood metabolites at the mRNA level.

## Results

### Analysis of genetic correlation between pulmonary embolism and human blood metabolites

**Fig. 1:**
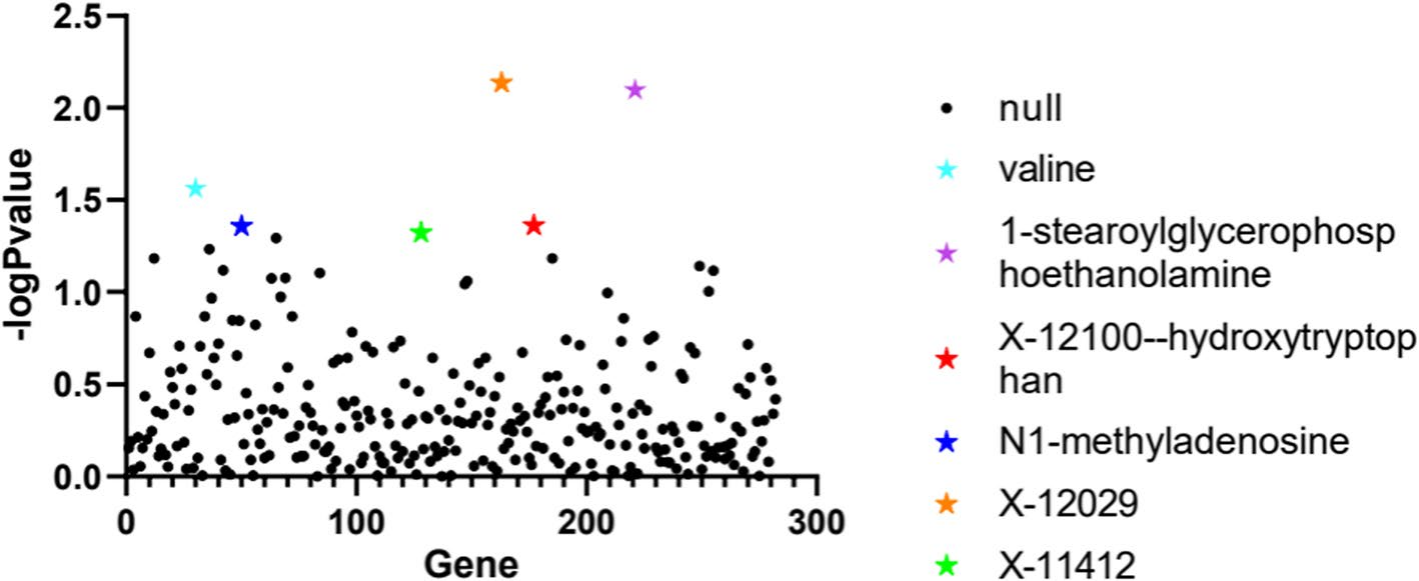
Scatter plot of the results obtained by genetic correlation analysis between pulmonary embolism and human blood metabolites. A scatter plot was used to analyze the genetic correlation between pulmonary embolism and human blood metabolites. Each dot represents a blood metabolite. The X-axis represents the blood metabolites, and the Y-axis represents the -log of the *p*-value of the analysis results

We found 6 blood metabolite genes associated with pulmonary embolism, including 1-stearoylglycerophosphoethanolamine (correlation coefficient = 0.0047, *p*-value = 0.008), x-1210-hydroxytryptophan (correlation coefficient = 0.0045, *p*-value = 0.0435), x-12,029 (correlation coefficient = 0.0134, *p*-value = 0.0073),× 0.0134, *P*-value = 0.0073), x-11,412 (correlation coefficient = 0.1288, *P*-value = 0.0476), N1-methyladenosine (correlation coefficient = 0.185, *P*-value = 0.0439), and Valine (correlation coefficient = 0.1569, *P*-value = 0.0274) (Table 1, Fig. 1).

**Table 1:** Genetic correlation between human blood metabolites and pulmonary embolism (*P* value < 0.05)

	Blood metabolites	Gene	Genetic Correlation	*p* values
pulmonary embolism	1-stearoylglycerophosphoethanolamine	LIPC	0.0047	0.008
	X-12100--hydroxytryptophan	USE1	0.0045	0.0435
	N1-methyladenosine	SPATA20	0.185	0.0439
	valine	GYPA	0.1569	0.0274
	X-12029	CRISP2	0.0134	0.0073
	X-11412	AT1B2	0.1288	0.0476

GWAS data for pulmonary embolism were derived from a European cohort study of 448,312 pulmonary embolism cases and 3952 controls. GWAS data for human blood metabolites were also derived from a European cohort study involving 529 metabolites in plasma or serum of 7824 adults. We used LD score regression software (https://github.com/bulik/ldsc) to complete genetic correlation analysis between pulmonary embolism and blood metabolites

### Causal relationship between pulmonary embolism and human blood metabolites

**Fig. 2:**
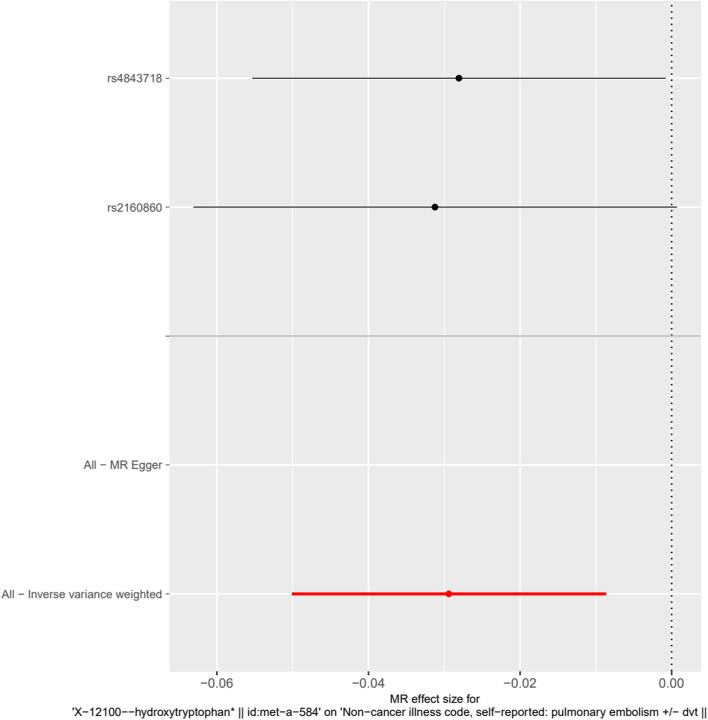
Forest map of the causal relationship between X-12100-hydroxytryptophan-associated SNPs and pulmonary embolism. Causality between X-12100-- Hydroxy Tryptophan) and Pulmonary embolism (outcome) was analyzed using an IVW model by MR analysis, and a significant causal relationship was found (β = − 0.0294, Se = 0.01056, *P* = 0.005349)

A significant causal relationship between x-12,100-hydroxytryptophan (exposure) and pulmonary embolism (outcome) was found by MR investigation according to IVW (β = − 0.0294, SE = 0.01056, *P* = 0.01056) (Table 2, Fig. 2).

**Table 2:** The results of causal analysis of human blood metabolites (exposure) and pulmonary embolism (outcome)

Exposure group	Number of SNP	Analytical method	Beta	SE	95%CI	P
1-stearoylglycerophosphoethanolamine	1	Wald ratio	−0.009597	0.007869	(−0.0250, 0.0059)	0.2226
X-12100--hydroxytryptophan	2	IVW	−0.0294	0.01056	(−0.0087, − 0.0500)	0.005349
X-11412	1	Wald ratio	−0.01947	0.0178	(−0.0543,0.0154)	0.274
X-12029	1		NA			
N1-methyladenosine	1	Wald ratio	−0.006814	0.0306	(−0.0668,0.0532	0.8238
valine	1	Wald ratio	−0.02911	0.02328	(−0.0747,0.0165	0.211

Tests were considered statistically significant at *P* values < 0.05. The MR base platform (http://app.mrbase.org/) was used for MR analyses

MR analysis showed no critical causal relationship between pulmonary embolism and the other five metabolites, and detailed results are presented in subsequent tables of this paper (Table 2, Table 3).

**Table 3:** Results of causal analysis of pulmonary embolism (exposure) and human blood metabolites (outcome)

Exposure group	Outcome group	Methods	Beta	SE	95%CI	*P* value
pulmonary embolism	1-stearoylglycerophosphoethanolamine	MR Egger	− 0.01012	2.555	(− 5.0179,4.9977)	0.9971
		Weighted median	−0.02833	0.9651	(−1.9199,1.8633)	0.9766
		Inverse variance weighted	0.2539	0.8356	(−1.4192,1.8917)	0.7612
pulmonary embolism	X-12100--hydroxytryptophan	MR Egger	−1.129	1.59	(−4.2454,1.9874)	0.5288
		Weighted median	−0.3097	0.6186	(−1.5222,0.9028)	0.6166
		Inverse variance weighted	0.08013	0.5019	(−0.9036,1.0639)	0.8732
pulmonary embolism	X-12029	MR Egger	−1.365	1.078	(−3.4779,0.7479)	0.2946
		Weighted median	0.007778	0.4192	(−0.8139,0.8294)	0.9852
		Inverse variance weighted	0.3129	0.4308	(−0.5315,1.1573)	0.4675
pulmonary embolism	X-11412 subunit beta-2	MR Egger	−1.613	1.609	(−4.7666,1.5406)	0.3899
		Weighted median	−0.456	0.6179	(−1.6671,0.7551)	0.4605
		Inverse variance weighted	−0.1042	0.5212	(−1.1258,0.9174)	0.8415
pulmonary embolism	N1-methyladenosine	MR Egger	0.1411	0.9921	(−1.8034,2.0856)	0.8959
		Weighted median	−0.3065	0.3746	(−1.0407,0.4277)	0.4133
		Inverse variance weighted	−0.3298	0.3075	(−0.9325,0.2729)	0.2835
pulmonary embolism	valine	MR Egger	−0.2964	1.072	(−2.3975,1.8047)	0.8002
		Weighted median	0.4962	0.3983	(−0.2844,1.2769)	0.2129
		Inverse variance weighted	0.5772	0.3468	(−0.1025,1.2569)	0.09607

Tests were considered statistically significant at *P* values < 0.05. Horizontal lines denote 95% confidence intervals. The MR base platform (http://app.mrbase.org/) was used for MR analyses

### Screening for differentially expressed genes associated with pulmonary embolism

**Fig. 3:**
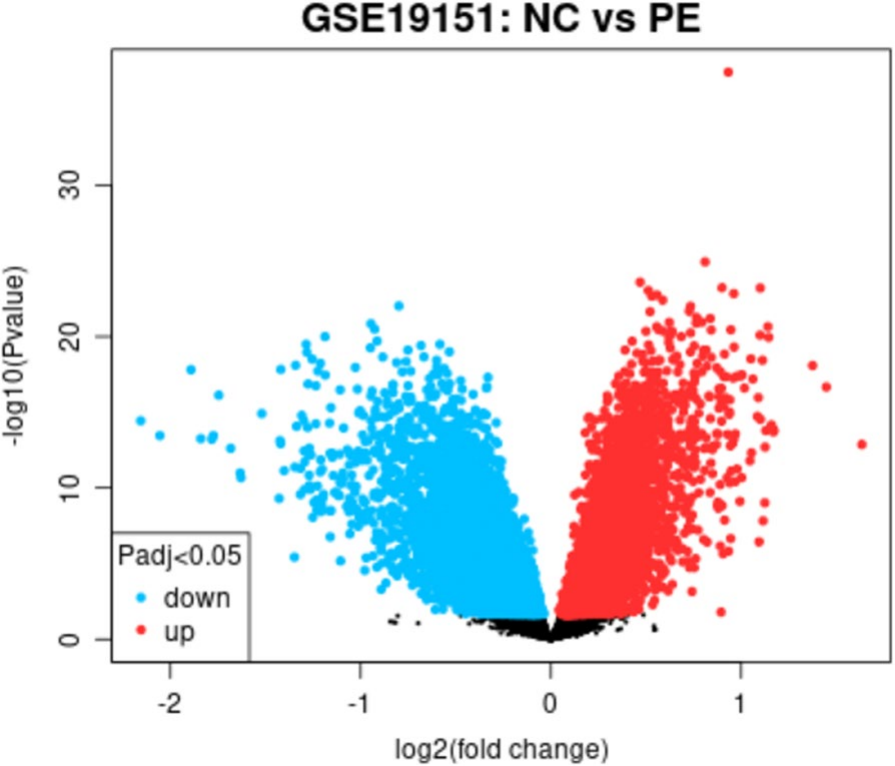
Heat map of differentially expressed genes between patients with pulmonary embolism and healthy control individuals. GSE19151, a dataset of microarray expression profiles downloaded from GEO, contained 10 pulmonary embolism samples and 7 healthy subjects. To analyze pulmonary embolism and differentially expressed genes (DEGs) among healthy people, we used GEO2R as a GEO analysis tool (https://www.ncbi.nlm.nih.gov/geo/geo2r/). We output the results into a heat map, where blue represents upregulated expression and green represents downregulated expression

The GSE19151 dataset contained sequencing data from 71 patients with pulmonary embolism and 62 healthy control subjects. After screening DEGs by GEO2R, 17,362 upregulated genes and 4854 downregulated genes were identified. Compared with six blood metabolites, two types of blood metabolite genes were found to be associated with pulmonary embolism, namely, 1 stearoylglycerophosphoethanolamine (LIPC) and X-12100--hydroxytryptophan. Genes for valine were not associated with pulmonary embolism severity, and the other blood metabolites, x - 12,029 and - 11,412, have not yet been named (Fig. 3, Table 4).

**Table 4:** Differential expression of genes encoding six blood metabolites proteins in pulmonary embolism

Plasma protein	Gene	*P* value	LogFC
1-stearoylglycerophosphoethanolamine	LIPC	0.696	0.022751
X-12100--hydroxytryptophan	IDO1	0.0337	−0.210549
N1-methyladenosine	NAT2	0.0025	0.0978175

GSE19151, a dataset of microarray expression profiles downloaded from GEO, contained 70 pulmonary embolism samples and 53 healthy subjects. To analyze pulmonary embolism and differentially expressed genes (DEGs) among healthy people, we used GEO2R as a GEO analysis tool (https://www.ncbi.nlm.nih.gov/geo/geo2r/)). We then looked for genes encoding six blood metabolites in DEGs

## Discussion

First, we performed LDSC analysis of the GWAS data on pulmonary embolism and human blood metabolites and found that there were six blood metabolites that had relevant correlations with pulmonary embolism: glycerophosphatidylethanolamine, hydroxytryptophan, methyladenosine, and valine. x-12,029 and x-11,412 are also genetically associated with pulmonary embolism, but they have not yet been named, so we have not discussed them in this paper. We then analyzed the causal relationship between these six blood metabolites and pulmonary embolism and found that only hydroxytryptophan was causally associated with pulmonary embolism, while the other five blood metabolites were not significantly causally associated with pulmonary embolism. Finally, we compared the six blood metabolite genes obtained with the differentially expressed genes of pulmonary embolism and found that LiPC and NAT2 were differentially expressed in patients with pulmonary embolism when compared to the normal healthy population.

5-HTP is a naturally occurring amino acid (AA) that is not involved in protein synthesis. It is derived from tryptophan (Trp), whose 5′-hydrogen atom on the phenyl ring is substituted with a hydroxyl group.9 5-HTP plays an important role in the regulation of mood, behavior, sleep, pain, body temperature, and other physiological functions [31]. It has been shown that 5-HTP is associated with depression [32], and the intake of 5-hydroxytryptophan results in a significant increase in the level of 5-HTP acid (a breakdown product of 5-HTP) in the cerebrospinal fluid (CSF), which improves the symptoms of depressed patients. It has also been shown that serotonin is associated with obesity and that the peripheral synthesis of 5-HTP influences human obesity by controlling vasoconstriction, intestinal motility and glucolipid metabolism through interaction with 5-HTP receptors. Our study suggests a genetic correlation between 5-HTP and pulmonary embolism complications after bone fracture surgery, and 5-HTP expression differs between pulmonary embolism patients and normal subjects. However, it has not yet been investigated whether 5-HTP plays a role in the complications of pulmonary embolism after bone fracture surgery.

n1-Methyladenosine (M1A) is a methylation modification of RNA that has attracted attention because it plays different roles in different biological processes. n1-Methyladenosine plays an important role in cell differentiation, protein production and bioregulation, and some studies have shown that n1-methyladenosine may play a key role in regulating HCC processes [33]. n1-Methyladenosine dysregulation can affect several biological processes, including cell proliferation, self-renewal programs, and apoptosis [33]. It was reported that n1-methyladenosine showed a gradient in superior vena cava pulmonary artery plasma, suggesting a potential relevance of metabolism [34], while an association was also found between sexuality and chronic thromboembolic pulmonary hypertension (CTEPH) [35]. The results of this study were consistent with previous reports, further validating the importance of the present study.

Valine is one of the 20 amino acids that make up proteins and is an essential mammalian glycogenic amino acid. Studies have shown that obese and overweight children have higher plasma valine concentrations than normal weight children [36], suggesting that this may be related to obesity and diabetes. Another study has shown that valine is essential for effective protein cross-linking, which suggests that valine is associated with clotting time.12 This also confirms the results of the present study and makes it more convincing.

It has been shown that 1-stearoylglycerophosphoethanolamine is associated with lipid abnormalities and liver function in a nonhuman primate model of the plague [37]. No other studies on 1-stearoylglycerophosphoethanolamine were found, so its association with pulmonary embolism, if biological, still needs further study.

### Strengths and limitations

Our study is the first to use GWAS data and related studies about pulmonary embolism and human blood metabolites. We analyzed whether there is a genetic correlation between pulmonary embolism and blood metabolites and demonstrated that blood metabolites with abnormal expression play a biological role in the development of pulmonary embolism. Finally, we verified whether there was differential expression of metabolite genes associated with pulmonary embolism in the blood of patients with pulmonary embolism when compared to normal healthy subjects, and we obtained meaningful evidence. These results provide clues to further investigate the genetic mechanisms of pulmonary embolism and blood metabolites after bone fracture surgery and provide new clinical ideas for the prevention of postoperative complications of pulmonary embolism. Nevertheless, there are some limitations of our study. First, genome-wide association studies on pulmonary embolism and data on human blood metabolites were conducted in European populations, which may limit the generalization of the findings to all people and lack some convincing power. Second, as mentioned above, the causal analysis using MR relied on three assumptions that were not always fully satisfied. Since we identified only two SNPs, it cannot do MR sensitivity analysis, and thus the analysis of horizontal pleiotropy was lacking, thus our results should be used cautiously when studying blood metabolites in pulmonary embolism. At the same time, rs4843718 in this study is related to many phenotypes, and there may be horizontal pleiotropy affecting the results, which we will continue to study in the follow-up study. At the same time, because the British Biobank was selected for this study data, so there is a certain selection bias. At the same time, there may be potential correlations between the human blood metabolites in this study, which will also have a certain impact on our results. In this study, we considered large-scale GWAS data of other blood metabolites, but due to the difficulties in data integration due to the different types of metabolites, only the GWAS data of Shin et al. were used in this paper, and the sample size of metabolites was not To achieve optimality, we will analyze the large GWAS data of the remaining blood metabolites in follow-up studies. Finally, the conclusions drawn from the analysis have not been validated by molecular biology or biochemistry experiments, so we will validate the findings of this paper in subsequent studies. In the meantime, we encourage our colleagues to continue their research in accordance with the new ideas presented in this paper.

## Conclusion

We used GWAS data to analyze pulmonary embolism and blood metabolites in a European population. LD score regression analysis suggested that six blood metabolites were found to be genetically associated with pulmonary embolism, while hydroxytryptophan was found to be causally associated with pulmonary embolism. In addition, genes encoding 1-stearoylglycerophosphoethanolamine and N1-methyladenosine were differentially expressed in patients with pulmonary embolism and present in normal healthy individuals. This finding provides new ideas for future studies on the genetic mechanisms, biomarkers diagnosis and treatment of pulmonary embolism after bone fractures and also provides insights into the clinical prevention of pulmonary embolism complicated by bone fracture.

## Data Availability

The datasets analyzed during the current study are available from the Gene Expression Omnibus database (https://www.ncbi.nlm.nih.gov/gds) accession number: GSE19151; the UK biobank (http://geneatlas.roslin.ed.ac.uk/) fields: 20002.; The datasets used and/or analyzed in the remaining studies are available upon reasonable request to the corresponding authors.
